# Clinical efficacy of percutaneous intramedullary aspiration, irrigation, and injection of absorbable bone in pediatric simple bone cyst

**DOI:** 10.3389/fped.2025.1672550

**Published:** 2025-09-10

**Authors:** Gang Xu, Wei Sun, Shiquan Zhang, Wei Li

**Affiliations:** Department of Musculoskeletal Tumor Surgery, First Affiliated Hospital of Shenzhen University, Shenzhen Second People’s Hospital, Shenzhen, China

**Keywords:** simple bone cyst, children, recurrence, minimally invasive surgery, fracture

## Abstract

**Background:**

Simple bone cysts (SBCs) are common benign bone lesions that primarily affect the long bones of children and adolescents. Due to their tendency to recur and their potential to cause pathological fractures, a range of treatment strategies has been investigated. This study evaluates the clinical efficacy of percutaneous intramedullary aspiration, irrigation, and injection of absorbable bone (PAIB) in the treatment of pediatric SBCs.

**Methods:**

All patients underwent the PAIB procedure. Postoperative evaluation involved radiographic assessment to monitor recurrence and identify potential complications, with magnetic resonance imaging (MRI) performed when clinically indicated.

**Results:**

In this cohort of 36 patients with SBC treated using the PAIB technique, the mean follow-up duration was 33.5 months (range: 12–66 months). Recurrence occurred in 6 patients (16.6%), including one case complicated by a pathological fracture. Five of the recurrent cases underwent repeat PAIB procedures, resulting in complete resolution in two patients. Postoperative imaging revealed small residual cysts in three cases. Bone healing was successfully achieved in 32 patients. Graft bone exudation, identified as a potential risk factor for recurrence, was observed in seven patients. All patients exhibited satisfactory functional outcomes throughout the follow-up period.

**Conclusion:**

PAIB appears to be a safe, effective, and minimally invasive treatment option for the management of pediatric simple bone cysts.

## Introduction

Simple bone cyst (SBC) is a benign bone lesion that predominantly affects children and adolescents. While SBCs can develop in various skeletal locations, they most frequently involve the metaphyseal regions of long bones, particularly the proximal humerus, proximal femur, and calcaneus (1). Although the exact pathogenesis remains uncertain, current literature suggests that SBC may result from vascular obstruction during bone growth, leading to the formation of fluid-filled cavities within the bone. This pathological process compromises bone integrity by reducing cortical thickness and causing cortical expansion, thereby increasing the risk of pathological fractures, even following minor trauma (2). The primary objective of treatment is, therefore, to prevent or manage such fractures. Asymptomatic lesions with minimal bone destruction or low fracture risk may be managed conservatively with regular radiographic monitoring. However, certain cysts may enlarge over time, further weakening cortical structure and elevating fracture risk (3). Current treatment approaches include cyst aspiration and injection, decompression techniques, and various surgical interventions (2, 4). Although the local recurrence rate and complications remain inevitable, minimally invasive treatments have become increasingly popular in the management of SBC due to their potential advantages, including reduced recurrence and faster recovery.

In this study, we present a minimally invasive treatment technique for SBC in children that involves percutaneous intramedullary aspiration, irrigation, and injection of absorbable bone (PAIB). The objectives of this study are (1) to examine the prognostic outcome and complications in patients treated with PAIB, and (2) to conduct a literature review to compare the clinical outcomes and complications of various reconstruction methods.

## Methods

This retrospective study included a total of 36 pediatric patients (13 females and 23 males) with non-pathological fractures with SBC who underwent PAIB between 2019 and 2024 at the First Affiliated Hospital of Shenzhen University. All patients were followed for a minimum of 12 months. Twenty-one SBCs were located in the proximal humerus, fourteen were in the proximal femurs, and one was in the proximal fibula. The mean age at the time of treatment was 11 years (range: 4–17 years), and the mean follow-up duration was 33.5 months (range: 12–66 months). Informed consent was obtained from all patients and/or their legal guardians. The study was approved by the Ethics Committee of Shenzhen University.

### Follow-up and outcome measures

All patients underwent preoperative imaging, including x-ray, computed tomography (CT), or magnetic resonance imaging (MRI). Pathological biopsy was performed when clinically indicated. Surgical indications included pain, cortical bone thinning, or a high risk of pathological fracture. All procedures were performed by experienced senior surgeons from the same team. Routine postoperative assessments involved anteroposterior and lateral radiographs at 1, 3, 6, 12 months and then every.

6 months up to 5 years, with MRI added if suspect recurrence. Radiographic outcomes were evaluated according to the criteria described by Capanna (5), where Grade I indicates complete healing; Grade II, the presence of residual lesion; Grade III, recurrence defined by the reappearance of radiolucent areas within the original cyst cavity or cortical thinning; and Grade IV, no response to treatment.

### Surgical procedure

**Figure 1:**
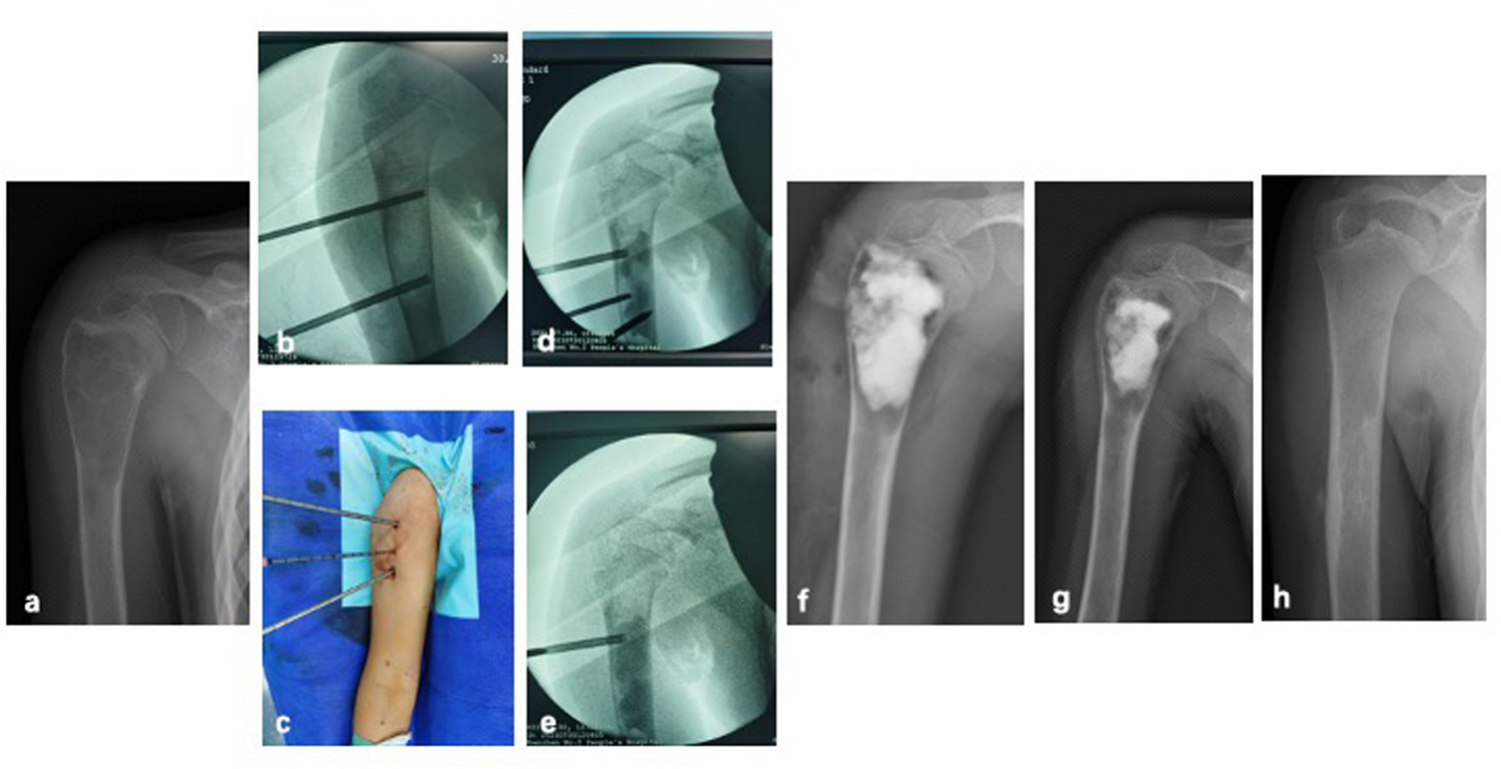
A 8-year-old female with SBC of right humerus treated by PAIB. **(a)** Radiograph of preoperative x-ray. **(b)** Percutaneous punctures were peformed by puncture needles, and inserted intramedullary into both ends of the lesion. **(c)** Additional puncture can be performed in the center of the lesion. **(d,e)** Absorbable artificial bone graft material was injected into the cavity under C-arm guidance. **(f)** Immediate postoperative radiograph. **(g)** 1-month postoperative radiograph. **(h)** Postoperative radiograph after operation 6 months showed SBC healed.

**Figure 2:**
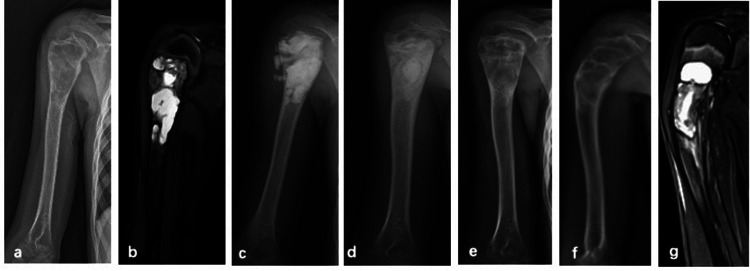
A 11-year-old male with SBC of left humerus treated by PAIB. **(a,b)** Radiograph of preoperative x-ray and MRI scan. **(c)** Immediate postoperative radiograph showed exudation of bone graft. **(d)** 3-month postoperative radiograph, the exudation of bone graft was absorbed. **(e)** 9-month postoperative radiograph showed recurrence was suspected. **(f,g)** Pathologic fracture occurred after operation 12 months by x-ray and MRI scan.

C-arm fluoroscopy was utilized to precisely localize the SBC lesion. Percutaneous punctures were performed with puncture needles (Bone Marrow Aspiration Biopsy Needle, Biomid Ⅱ, 9G, Gallini, Italy) inserted intramedullary at both ends of the lesion. For lesions exceeding 5 cm in length, an additional puncture was made at the center of the lesion. The cyst membrane was repeatedly disrupted using the puncture needle to facilitate cavity clearance. Subsequently, the intramedullary cavity was thoroughly lavaged with a large volume of saline, and residual fluid along with detached cyst membrane fragments were aspirated. Under continuous C-arm guidance, absorbable artificial bone graft material was then injected into the cavity (The dosage depends on the cavity). Two types of absorbable graft materials were employed: Pro-Dense Injectable Regenerative Graft (calcium sulfate and calcium phosphate matrix mixed with beta-tricalcium phosphate, 15cc), Wright Medical Technologies®, Memphis, USA and NovaBone Putty® Syringe Bioactive Synthetic Bone Graft (bioactive calcium phosphosilicate, 10cc), USA (Figure 1). Graft bone exudation is defined as absorbable artificial bone graft material was exudated from needle tract after injection (Figure 2c).

### Statistical analysis

The Fisher's exact test was used to evaluate potential risk factors for SBC recurrence, including gender, age, tumor length, lesion location, distance from the physis, and bone graft exudation. Statistical analyses were conducted using IBM SPSS Statistics version 24.0. A *p*-value less than 0.05 was considered statistically significant.

## Results

**Figure 3:**
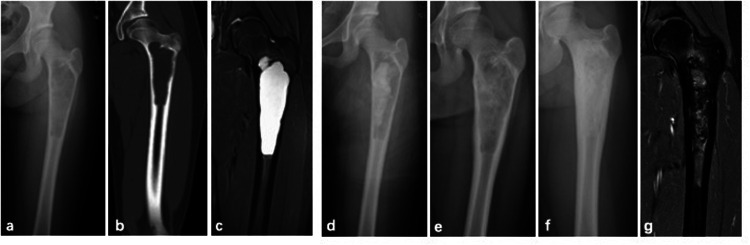
A 11-year-old female with SBC of left femur treated by PAIB. **(a)** Radiograph of preoperative x-ray. **(b,c)** CT and MRI scan. **(d)** Immediate postoperative radiograph. **(e)** 3-month postoperative radiograph. **(f)** 12-month postoperative radiograph showed SBC is healed. **(g)** MRI scan after operation 12 months.

At the final follow-up, recurrence was observed in 6 of the 36 patients. One patient developed a fracture 12 months postoperatively and subsequently underwent surgery involving curettage combined with bone grafting (Figure 2). Five patients received repeat PAIB treatment, with two achieving complete healing. Three patients exhibited persistent small residual cysts postoperatively (Capanna Grade II); among them, one patient was managed conservatively and showed no change at the latest one-year follow-up. Two other patients underwent repeat surgical treatment with PAIB and currently show no evidence of recurrence at the latest follow-up. Overall, successful bone healing was achieved in 32 patients (Figure 3). Postoperative graft bone exudation was noted in seven patients but resolved spontaneously within 3.5 months. No cases of limb dysfunction, nerve injury, heterotopic ossification, surgical site infection, or epiphyseal damage were observed at the final follow-up (Figure 2).

Subgroup analysis demonstrated a statistically significant difference in recurrence rates between patients with and without bone graft exudation (*P* < 0.05) (Table 1).

**Table 1:** Association between variables and recurrence.

Variable	Number	Recurrence	*P*-value
Age, year			
<10	11	3	0.58
≥10	25	3	
Length of tumor, cm			
<5	22	2	0.11
≥5	14	4	
Gender			
Male	23	4	0.55
Female	13	2	
Location			
Humers	21	3	0.35
Femur	14	3	
Contact with phsis			
Yes	12	4	0.11
No	24	2	
Effusion of bone graft			
Yes	7	4	0.042
No	29	3	

## Discussion

As early as 1979, Scaglietti et al. reported that intralesional injection of methylprednisolone acetate (MPA) could effectively treat SBCs with minimal invasiveness, thereby avoiding the risks associated with open surgery. However, the reported non-union rates ranged widely from 10% to 75%. To enhance bone healing, some clinicians combined autologous bone marrow and/or demineralized bone matrix with steroid injections, but multiple treatments were often required to achieve complete resolution (6–8). A comparative study involving 69 pediatric SBC cases treated by curettage and bone grafting, steroid injection, or decompression using cannulated screws reported initial efficacy rates of 25%, 12%, and 29%, respectively, after a mean follow-up of 69 months. Following a second treatment, healing rates improved to 50%, 19%, and 65%, respectively, suggesting that decompression and curettage may be more effective than steroid injection (9). Although cyst membrane curettage, alone or combined with bone grafting, has long been considered a cornerstone of SBC treatment, reported healing rates remain below 50% (10). Zhang K et al. reported that open curettage combined with bone grafting resulted in complete healing in only 30% of cases, with local recurrence rates ranging from 15% to 40%, and a postoperative fracture rate of 16% (11) (Table 2). Additionally, some studies have suggested that combining curettage with cryotherapy may reduce recurrence rates (12). However, this approach is generally not recommended as first-line treatment due to the risk of postoperative cortical weakening, which can predispose patients to fractures (11, 13). Currently, minimally invasive techniques are increasingly adopted for SBC treatment. For example, Aiba et al. reported on 37 patients treated arthroscopically, allowing for direct visualization and less invasive lesion removal, which resulted in a recurrence rate of 7 patients and a postoperative fracture rate of 5%. Although post-curettage cortical weakening may increase fracture risk, the overall prognosis with arthroscopic treatment appears favorable compared to open surgery (14).

**Table 2:** Published studies of treatment methods for SBC.

Author	Method (number of paients)	Year of publication	Number of patients	Gender (num)	Age (years)	Site (num)	Follow-up period (months)	Recurrence and no response	Healing rate	Postoperative fracture	Union time (months)	Infection(%)	Others(number)	Associated with recurrence or failure
Roposch A. et al. (24)	EIN	2000	32	Male: 25 Female: 7	9.8	Humerus: 21 Femur: 9 Radius: 1	53.7	6%	93.7% (Capanna I: 43.7%)	N/A	3–105	N/A	Change of nails (9)	
Glanzmann MC. et al. (20)	EIN	2007	22	Male: 15 Female: 7	9	Humerus: 16 Femur: 6	24	9%	90.9% (Capanna I: 72.7%)	0	16	27.20%	Removed implants	
Brecelj J. et al. (9)	OCBG Steroids injection -Cannulated screw insertion	2007	69	Male: 46 Female: 23	9.8	Humerus: 55 Femur: 9 Fibula:2 Radius: 1 Calcanues:1 Tibia:1	69	N/A	First OP: 25%, 12%, 29% Second OP: 50%, 19%, 65%	N/A	N/A	N/A		Site involving the humerus
Sung AD. et al. (13)	Steroids injection (94) OCGB (39) SABMI (34)	2008	167	Male: 120 Female: 47	9.5	Humerus: 115 Femur: 42	88	N/A	16% 36% 50%	18% 2.6% 12%	N/A	N/A	Occasional pain or numbness 5.3%, 21%, 8.8%	Younger age Treatment with steroids
Hou HY. et a. (17)	Steroid and autogenous bone-marrow injection (9) OCBG without instrumentation(12) OCBG with internal instrumentation (7) MIC + ethanol cauterization + cannulated screw (12)	2011	40	Male: 30 Female: 10	13.2	Humerus: 28 Femur: 9 Fibula:1 Radius: 1 Tibia:1	18–84	55% 33% 14% 8%	33.3% 66.7% 85.7% 91.6%	11% 0 0 8%	3.7 ± 2.3 23.4 ± 14.9 12.2 ± 8.5 6.6 ± 4.3	N/A	Mean number of surgery	Steroid and autogenous bone-marrow injection
Tena-Sanabria. et al. (12)	OCBG + Cryotherapy	2014	12	Male: 8 Female: 4	9.5	Humerus: 7 Tibia: 3 Pelvis: 1 Clavicle: 1	12–36	0	75%	0	N/A	N/A		
Shirai T. et al. (18)	PC + Cannulated hydroxyapatite pin	2015	43	Male: 35 Female: 8	12.1	Calcanues: 23 Humerus: 15 Femur: 3 Pelvis: 2	26.6	11.60%	88%	0	6.4	13%		Age <10 years Site involving the humerus Contact with the growth plate
Li.WC. et al. (22)	EIN (23) Injection of ABM (23)	2016	46	Male: 17 Female: 29	9	Humerus: 30 Femur: 16	62	ABM: 13.0%	69.6% 60.9%	4.3% 17.3%	N/A	4.3% 13%	EIN:Skin irritation(3)	
Aiba H. et al. (14)	PC + Endoscopic curettage	2018	37	Male: 24 Female: 13	14.7	Humerus: 18 Femur: 8 Calcanues: 6 Pelvis:2 Radius:2 Tibia:1	33.8	18.90%	83.7%	5%	4	N/A	Transient radial nerve palsy (1)	Contact with the physis
Higuchi T. et al. (19)	PC + Cannulated hydroxyapatite pin (39) OCGB (calcium phosphate cement) (36)	2018	75	Male: 50 Female: 25	17.5 ± 11.6	Calcanues: 24 Humerus: 23 Femur: 8 Pelvis and spine: 13 Tibia: 2 Talus: 2 Ulna: 1	39.5 26.4	24.2% 12.7%	N/A	0	5.5 ± 3.1	N/A		Age <10 years Long bone cysts Active phase cysts
Zhang K. et al. (11)	EIN + CBG (30) OCBG(32)	2019	62	Male: 43 Female: 19	8.2	Humerus: 27 Femur: 35	27	10% 31.2%	90.0% (Capanna I: 56%) 68.8% (Capanna I: 31.2%)	OCBG: 16.6%	10.6 ± 6.8 18.4 ± 13.4	EIN + CBG: 3%		
Mavcic B. et al. (16)	Drainage screws -EIN CBG	2019	106	Male: 74 Female: 32	10.3	Humerus:106	68.4	Mean: 1.1:0.5:1.8	36%:32%:24%	Mean: 0.4:0.2:0.5	N/A	N/A	Requre three surgical procedures	Drainage screws
Zhou JW. et al. (23)	EIN + MPA (24) EIN + CBG (29)	2021	53	Male: 39 Female: 14	7.6 8.1	Humerus: 30 Femur: 23	>36	8.3% 20.6%	91% (Capanna I: 33%) 79% (Capanna I: 37%)	0	6–8	EIN + CBG: 10%	Injection times 1.8 (1–3)	
Xu G. et al.	PAIB	2025	36	Male: 23 Female: 13	11	Humerus: 21 Femur: 14 Febula: 1	33.5	16.6%	83.30%	1	N/A	0%	Graft bone exudation (7)	Graft bone exudation

PC, percutaneous curettage; ABM, injection of autogenous bone marrow; EIN, elasticstable intramedullary nailing; MPA, methylprednisolone acetate; CBG, curettage, bone grafts; SABMI, steroid and autogenous bone-marrow injection; OCBG, open curettage, bone graft; MIC, minimally invasive curettage; PAIB, percutaneous aspiration, irrigation, injection of absorbable bone; N/A, not avalible.

Cannulated screws and elastic intramedullary nails (EIN) have demonstrated the ability to provide continuous decompression of SBCs, resulting in satisfactory therapeutic outcomes. However, comparative studies have indicated that cannulated screws yield lower healing rates compared to other surgical interventions and often require secondary surgery for hardware removal (9, 15, 16). In contrast, Hou HY et al. reported that percutaneous minimally invasive procedures using cannulated screws achieved significantly higher bone healing rates than open curettage and steroid injection (17). Shirai et al. described 43 cases treated with continuous decompression via cannulated hydroxyapatite (HA) pins, which offer the advantage of continuous lesion decompression without necessitating hardware removal. Recurrence occurred in only 5 cases, with 2 patients exhibiting persistent small residual cysts (18). Higuchi et al. found that HA pins provide superior healing rates compared to calcium phosphate cement (CPC) filling but are associated with higher relapse rates, potentially due to factors such as patient age under 10 years, lesion location in long bones, or proximity to the epiphysis (19). Glanzmann et al. reported a 90.9% efficacy rate in 22 SBC patients treated with EIN, with only two recurrences and four cases of residual lesions (20). Journeau et al. conducted a comparative study of steroid injection vs. intramedullary nail fixation, finding no significant difference in healing time but a higher complication rate in the steroid injection group, leading to the recommendation of EIN as the preferred treatment for weight-bearing bones (21). Li et al. demonstrated that both percutaneous intralesional injection of autologous bone marrow and EIN achieved satisfactory outcomes, with EIN showing significantly fewer complications (22). Zhou et al. compared outcomes between 24 children treated with EIN combined with MPA injection and 29 children undergoing curettage, bone grafting, and EIN fixation; the EIN + MPA group showed superior recurrence and efficacy rates, as well as better early postoperative functional activity, shorter hospital stays, reduced intraoperative blood loss, and shorter operation times (23). Whether used alone or in combination with MPA and autologous bone marrow (ABM), EIN achieves healing rates exceeding 70% and recurrence rates below 15%, providing favorable prognosis and immediate mechanical stability. Consequently, EIN is widely recommended as a primary treatment option (13, 15, 24). However, this technique requires secondary surgery for hardware removal and carries risks of superficial infection and the need for multiple injections.

This study evaluated the use of PAIB, which offers several advantages including minimally invasive surgery, preservation of bone tissue integrity, and reduced risk of pathological fractures and associated complications. Previous studies have suggested that multiple punctures of the cyst wall and/or disruption of the cyst membrane can lower recurrence rates by interrupting membrane continuity (20, 25). In our approach, deliberate disruption of the cyst membrane was performed to reduce recurrence; nonetheless, a recurrence rate of 16.6% was observed. Additionally, graft bone exudation at the needle tract was noted in some cases and was managed conservatively with observation rather than secondary surgical intervention. Follow-up imaging demonstrated gradual absorption of the exudated graft material, without evidence of heterotopic ossification or functional impairment. Subgroup analysis revealed a statistically significant difference in recurrence rates between patients with and without graft bone exudation. As the number of cases involving recurrence with graft bone exudation is relatively small, the underlying cause of the statistically significant difference is difficult to determine. It may be associated with the presence of residual cysts in certain localized areas. Bone healing time was not analyzed, as final healing was confirmed in all cases without SBC recurrence.

Although some SBC cases may spontaneously resolve following pathological fractures, such fractures can result in complications including mal-union, epiphyseal injury, limb length discrepancy, and growth plate disturbances (3, 14). The Cyst-Index is widely used to assess fracture risk; however, its predictive accuracy remains controversial. Beyond preventing pathological fractures, preventing recurrence is equally critical, as many recurrent cases require one or more additional surgical interventions. Minimally invasive surgeries are often preferred in pediatric patients due to their potential to reduce complications associated with repeated procedures. A meta-analysis encompassing 62 studies with a total of 3,217 SBC patients reported an overall recurrence or persistence rate of 23.9%. Conservative treatment accounted for 61.1% of cases, curettage with autologous bone grafting for 23.2%, and methylprednisolone acetate injections showed a recurrence rate of 28.5% (1). Comparative studies involving 77 patients managed conservatively and 64 undergoing surgical treatment demonstrated healing rates of 30% and 83%, respectively, indicating that surgical interventions are significantly more effective than observation alone (15, 26). Currently, various treatment modalities exist for SBC, with variable outcomes reported in the literature demonstrating acceptable recurrence and complication rates. Nevertheless, each treatment approach has inherent limitations, making direct comparison of their efficacy challenging.

Notably, an online survey of 444 surgeons from North America and Europe examined current practices in diagnosing and managing SBC. Among respondents, approximately 53% recommended surgical intervention for asymptomatic SBC cases with a high fracture risk, while 71% favored treatment for symptomatic (painful) SBC (27). These findings highlight that variability in initial diagnosis, treatment strategies, fracture risk assessment, and surgical techniques may contribute to differences in patient prognosis. Additionally, factors such as patient age under 10 years, lesion location within long bones, and proximity to the epiphysis have been identified as risk factors for SBC recurrence (7, 28, 29). Therefore, clinicians must carefully consider these variables when selecting treatment approaches to optimize outcomes.

This study has several limitations. First, as a retrospective single-center investigation, the sample size is relatively small. Second, the absence of a control group precludes direct comparison of recurrence rates, limiting our ability to definitively evaluate the efficacy of PAIB relative to other treatments; thus, our findings can only be contextualized against previously published studies. Consequently, the level of evidence provided is lower than that of randomized controlled trials. Third, the heterogeneity in lesion locations among patients may have influenced recurrence rates and complication profiles. To more comprehensively assess the safety and efficacy of this procedure, prospective, controlled studies with larger cohorts and long-term follow-up comparing multiple treatment modalities are warranted.

## Conclusion

The PAIB procedure demonstrates favorable clinical outcomes in the treatment of pediatric simple bone cysts, providing a safe, effective, and minimally invasive therapeutic option.

## Data Availability

The datasets presented in this study can be found in online repositories. The names of the repository/repositories and accession number(s) can be found in the article/Supplementary Material.
